# Downregulation of MIP-1α/CCL3 with praziquantel treatment in *Schistosoma haematobium *and HIV-1 co-infected individuals in a rural community in Zimbabwe

**DOI:** 10.1186/1471-2334-9-174

**Published:** 2009-10-23

**Authors:** RBL Zinyama-Gutsire, E Gomo, P Kallestrup, C Erikstrup, H Ullum, AE Butterworth, S Munyati, T Mduluza

**Affiliations:** 1 Biochemistry Department, University of Zimbabwe, Harare, Zimbabwe; 2 National Institute of Health Research, Ministry of Health and Child Welfare, Harare, Zimbabwe; 3 Department of Immunology, College of Health Sciences, University of Zimbabwe, Harare, Zimbabwe; 4 Centre of Inflammation and Metabolism, Department of Infectious Diseases 7641, Rigshospitalet, Blegdamsvej 9, 2100 Copenhagen, Denmark; 5 Department of Clinical Immunology, Aarhus University Hospital, Skejby Sygehus, Brendstrupgaardsvej 100, DK-8200 Aarhus N, Denmark; 6 Biomedical Research and Training Institute, Harare, Zimbabwe and London School of Hygiene and Tropical Medicine, London, UK

## Abstract

**Background:**

Chemokines have been reported to play an important role in granulomatous inflammation during *Schistosoma mansoni *infection. However there is less information on their role in *Schistosoma haematobium *infection, or on the effect of concurrent HIV-1 infection, as a potential modifying influence.

**Methods:**

To determine levels of MIP-1α/CCL3 chemokine in plasma of *S. haematobium *and HIV-1 co-infected and uninfected individuals in a rural black Zimbabwean community.

A cohort was established of HIV-1 and schistosomiasis infection and co-infection comprising 379 participants. Outcome measures consisted of HIV-1 and schistosomiasis status and levels of MIP-1α/CCL3 in plasma at baseline and three months post treatment. An association was established between MIP-1α/CCL3 plasma levels with HIV-1 and *S. haematobium *infections.

**Results:**

A total of 379 adults formed the established cohort comprising 76 (20%) men and 303 (80%) women. Mean age was 33.25, range 17 - 62 years. The median MIP-1α/CCL3 plasma concentration was significantly higher in *S. haematobium *infected compared with uninfected individuals (p = 0.029). In contrast, there was no difference in the median MIP-1α/CCL3 levels between HIV-1 positive and negative individuals (p = 0.631). MIP-1α/CCL3 concentration in plasma was significantly reduced at three months after treatment with praziquantel (p = 000).

**Conclusion:**

The results of our study show that the MIP-1α/CCL3 levels were positively associated with *S. haematobium *egg counts at baseline but not with HIV-1 infection status. MIP-1α/CCL3 levels were significantly reduced at three months post treatment with praziquantel. We therefore conclude that MIP-1α/CCL3 is produced during infection with *S haematobium*. *S. haematobium *infection is associated with increased MIP-1α/CCL3 levels in an egg intensity-dependent manner and treatment of *S. haematobium *is associated with a reduction in MIP-1α/CCL3.

## Background

Human immunodeficiency virus (HIV)subtype 1, affects over 25 million persons in sub-Saharan Africa, with most living without access to antiretroviral treatment [1,2]. An estimated 200 million persons are infected with schistosomes [3,4]. The number of persons co-infected with these diseases is not known but in some areas the prevalence of co-infected persons is estimated to be high [5-9]. In humans, infection with the trematode parasites, *S. mansoni *and *S. haematobium*, is followed, after 4-6 weeks, by the deposition of eggs in the liver and other body organs, leading to immune activation [10]. The impact of schistosomiasis on the progression of HIV in co-infected persons has been contradictory, with reports from uncontrolled studies showing little or no effects of schistosomiasis treatment [5-9]. Infection with HIV induces immune activation with high levels of circulating cytokines, such as tumor necrosis factor-alpha (TNF-α) [11,12], interleukin-6 (IL-6) [13,14], IL-10 [13,15,16], and some chemokines [14,15]. Activated CD4 cells express increased levels of the co-receptors CCR5 and CXCR4 speculated to enhance HIV entry and infect host cells [17], and MIP-1α/CCL3 enhances expression of these receptors that are also used by the virus. *In vitro*, the cytokines TNF-α and IL-6, as well as the chemokines IL-8 and MIP-1α/CCL3 can activate and assist the translocation of the transcription factor nuclear factor (NF)-κB in monocytes or macrophages [17]. In uninfected cells, this leads to cell proliferation and differentiation. However, the HIV provirus DNA carries multiple NF-κB binding sites; thus, the activation of NF-κB in HIV-infected cells results in viral nucleus material translocation into the host nucleus thereby undergoing replication [17]. Conversely, the anti-inflammatory cytokines IL-10 and TGF-β downregulate the pro-inflammatory cytokine TNF-α and MIP-1α/CCL3, thereby inhibits HIV replication *in vitro *in macrophages [17-19].

Schistosomiasis and some helminth infections are known to induce systemic inflammation associated with increased activation of CD4 cells and higher levels of CD8 cells [20], including surface receptor expression [21]. The initial acute schistosomiasis infection is characterized by production of T helper type 1 (Th1) cytokine that include interferon-γ and production of Th2 cytokines including IL-10 that would predominate in the subsequent early phases of the infection, attributed to play an important anti-inflammatory role [22-24]. There are few reports regarding levels of circulating cytokines during HIV and schistosomiasis co-infection, but Brown et al., (2005) reported that circulating IL-10 is reduced after treatment with praziquantel in co-infected persons and production of other cytokines during HIV-infection might also be affected by schistosomiasis co-infection [8,25-28]. Greater concentrations of MIP-1α/CCL3/have been reported in *S. mansoni *infected individuals compared to uninfected individuals [8]. Plasma levels of MIP-1α/CCL3 have also been investigated and reported in several studies of *S mansoni *infected humans, murine models [24] and cell culture infections [27,28], but none for *S. haematobium*. The aim of our study was to determine the plasma MIP-1α/CCL3 concentration in *S. haematobium *infected and uninfected humans at baseline and at three months after treatment with praziquantel and the influence of HIV-1 co-infection. We hypothesized that schistosomiasis would induce systemic inflammation indicated by the levels of circulating soluble MIP-1α/CCL3, a proinflammatory chemokine, and observed the effects of schistosomiasis treatment with praziquantel.

## Methods

### Setting and study population

The study was conducted from October 2001 to June 2003 in Mupfure and adjacent areas in Shamva District, Mashonaland Central Province, Zimbabwe. This rural area is characterized by subsistence farming, and the main source of water for irrigation, bathing, and washing is the Mupfure River, which is infested mainly by *Bulinus *species snails but also by *Biomphalaria *species snails [29]. No schistosomiasis control program has previously targeted the adult population of the area. The study population was composed of adults (>18 years old) residing in the area who were willing to submit urine, stool, and blood samples and to be tested for HIV-1. Recruitment of participants was achieved through community meetings and was facilitated by local village health workers. The Medical Research Council of Zimbabwe and the Central Medical Scientific Ethics Committee of Denmark approved the study, and informed consent was obtained from all participants. In addition, permission was given by the provincial medical director of Mashonaland Central Province, the district medical officer of Shamva District, and the village leaders.

### Screening procedure for HIV-1 serological and parasitological testing

Screening procedures were performed on all willing participants. HIV-1 testing was performed confidentially. Pretest and post-test counseling was provided in the participants' native language (Shona) by qualified personnel. In the field, a rapid HIV-1/2 test kit was used (Determine; Abbott Laboratories). All individuals who were initially found to be HIV-1 positive were retrospectively retested using a different rapid test kit (Oraquick by Orasure Technologies, Serodia by Fujirebio, or Capillus by Trinity Biotech). For participants subsequently included in the cohort, 2 ELISAs were performed on serum samples; No discrepancies were found between the results of the initial rapid HIV-1/2 test and those of the 2 subsequent ELISAs. Microscopic examination of fixed-volume urine samples filtered on Nytrel filters (VesterGaard Frandsen) was used to identify and quantitate eggs of *S. haematobium *by the syringe urine filtration technique [30]. Because of the diurnal and day-today variation in egg output, the urine samples were collected on 3 consecutive days [31]. The modified formol-ether concentration technique was used on 1 stool sample from each participant to detect eggs of *S. mansoni *and other helminth or parasites [32].

### Establishment of cohort

After the screening procedure, HIV-1-infected individuals who were coinfected with *Schistosoma *parasites were included in a prospective cohort. Simultaneously, a number of HIV-1-negative but schistosomiasis-positive individuals were included as controls, as were individuals infected only with HIV-1 and individuals with neither infection. The 379 participants included in the cohort were interviewed to obtain sociodemographic data and medical history, and a clinical examination was performed. Exclusion criteria were applied to participants presenting with clinical signs/symptoms of tuberculosis or severe anemia, but no participants were excluded for these reasons. Pregnant women were excluded from the study but were diagnosed and offered praziquantel as treatment for schistosomiasis after delivery and termination of breast-feeding.

### Blood sampling and MIP-1α/CCL3 assay

Blood was drawn into EDTA-coated tubes and kept cool until separation within a maximum of four hours after sampling. Plasma was transferred to cryotubes and stored in liquid nitrogen until shipment to laboratory, samples were stored at -80°C until analysis. Plasma levels of MIP-1α/CCL3 were assessed by an ELISA (Quantikine; R&D Systems, Minneapolis, MN) as described by the manufacturer [33]. Briefly, a double-sandwich ELISA in which MIP-1α/CCL3 was detected using a capture mouse anti human MIP-1α/CCL3 monoclonal antibody and a biotinylated goat anti-human MIP-1α/CCL3 antibody. The chemokine concentrations were determined in duplicate using a standard curve obtained from the known concentration of cytokine standards included in each assay plate within the range 15 pg/ml - 2000 pg/ml.

### Statistical analysis

All statistical analyses were performed using SPSS software (version 8.2). Egg counts were log-transformed to approximate normal distribution. Results from egg counts were used to stratify the schistosomiasis status of the participants into subgroups (no schistosomiasis, infection with *S. haematobium *only and co-infection with HIV-1. A 2-way analysis of variance (ANOVA), with HIV-1 status and schistosomiasis status as classifying variables, was used to identify differences between groups with respect to egg counts, age, and MIP-1α/CCL3, to explore the magnitude of the egg infection intensity effect on MIP-1α/CCL3. A *t *test was performed to evaluate differences in egg counts between HIV-1 groups and was complemented with an analysis of covariance (ANCOVA) to allow for adjustments according to age and sex. The magnitude of effects was evaluated by back transformation of the log-transformed difference in means and 95% confidence intervals (CIs) between groups. *P *< 0.05 was considered to be significant.

## Results

A total of 1545 individuals were screened by 3 consecutive urine samples, 1 stool sample, and information on HIV-1 status for recruitment of the 379 individuals into the cohort. Overall, 26.3% were HIV-1 positive, similar figure to the national prevalence levels [34]; 43.4% had schistosomiasis, and there were no differences in distribution of schistosome infections according to HIV-1 status (Table 1). The prevalence of other helminth infections was negligible and as reported previously [7,33,35]; very few individuals were diagnosed with *Taenia saginata *(*n *= 2), *Strongyloides stercoralis *(*n *= 2), or *Trichuris trichiura *(*n *= 1). Other identified intestinal parasites were the protozoans *Entamoeba histolytica *(*n *= 54; 47 [4.7%] were HIV-1 negative participants and 7 [1.7%] were HIV-1 positive participants; *P *< 0.05) and *Giardia lamblia *(*n *= 9; 8 were HIV-1 negative participants and 1 was an HIV-1 positive participant). Table 1 presents the sex and age distribution of the cohort. However, predominantly women participated in the study (75%), and there were no differences in sex distribution across the subgroups according to HIV-1 status or schistosomiasis status. Detailed results of the recruited individuals on interaction of schistosomiasis and HIV-1 are reported elsewhere [35]. The *t *test comparing urine egg counts confirmed no difference according to HIV-1 status for *S. haematobium *infected participants (*P *= 0.51; mean HIV-1 positive participants: HIV-1 negative participants, 0.91; 95% CI, 0.67-1.22). Of the 379 participants included in the prospective cohort, complete baseline information was available on all and the characteristics of the cohort population and the distribution of the schistosome and HIV-1 infections are presented in Table 1. There were no differences in egg counts attributable to either the interaction between HIV-1 and schistosomiasis or to HIV-1 status or schistosomiasis status separately.

**Table 1:** The characteristics of the study cohort in the *S. haematobium *and HIV-1 co-infection

Variable	n	Median MIP-1α/CCL3 (pg/ml)	(Interquartile range)	p-value
**Sex**				
Male	76	144	(65 - 295)	
Female	303	127	(56 - 205)	0.2130
Total	379			

**S. haematobium**				
Positive	263	171	(132 - 630)	
Negative	116	39	(4 - 90)	**0.0029**

**HIV status**				
Positive	198	124	(36 - 550)	
Negative	181	139	(47 - 445)	0.6312

**Age (years)**				
< 25	82	166	(55 - 525)	
≥ 25	297	121	(35 - 595)	0.2570

**Age groups (years)**				
< 20	24	146	(66 - 378)	
20 - 29	156	135	(78 - 402)	
30 - 39	92	113	(46 - 399)	
40 - 49	76	126	(72 - 332)	
50 +	31	163	(33 - 298)	0.5060

**Co-infection status**				
HIV+ S. haematobium +	154	144	(124 - 447)	
HIV- S. haematobium +	130	177	(133 - 506)	
HIV+ S. haematobium -	48	64	(6 - 79)	
HIV- S. haematobium -	47	30	(3 - 57)	**0.0008**

**CD4 vs MIP-1a/CCL3 in HIV+**				
Above 250	180	120	(38 - 440)	
Below 250	18	156	(36 - 398)	0.4993

**Eggs per 10 ml urine**				
0	116	36	(5 - 81)	
<10	177	80	(66 - 168)	
10 - <50	77	379	(211 - 680)	
>50	9	333	(199 - 862)	**0.0001**

**Time point**				
Baseline (before treatment)	263	131	(102 - 560)	
3 months post treatment	83	71	(6 - 94)	**0.0001**

Given are the medians and interquartile ranges of levels of plasma MIP-1α/CCL3 chemokine. The MIP-1α/CCL3 was measured in pg/ml using double sandwich ELISA within the range 15 pg/ml - 2000 pg/ml. Results from egg counts were used to stratify the schistosomiasis status of the participants into subgroups (no schistosomiasis, infection with *S. haematobium *only and co-infection with HIV-1. HIV-1 status and schistosomiasis status as classifying variables was used to identify differences between groups with respect to egg counts, age, and MIP-1α/CCL3; and *p *< 0.05 was considered to be significant.

**Figure 1:**
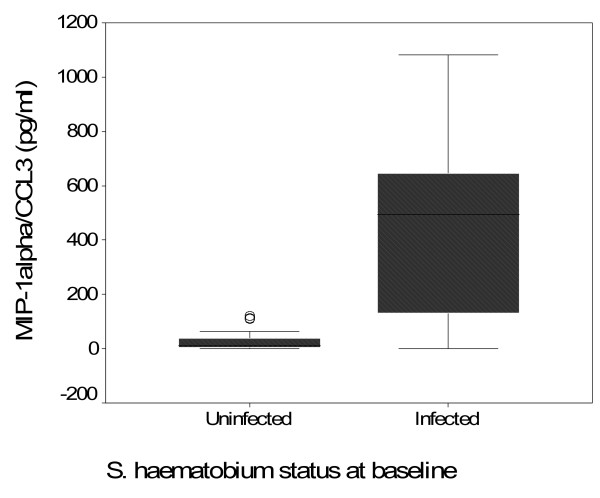
**Box-whisker plot of MIP-1α/CCL3 concentrations comparing *S. haematobium *infected and uninfected individuals**.

Plasma concentrations of MIP-1α/CCL3 were available on all 379 cohort participants for the baseline before treatment and on 83 selected at three months post treatment. The median MIP-1α/CCL3 plasma concentration was significantly higher in *S haematobium *infected compared with uninfected individuals (*S. haematobium *positive 171 pg/ml, *S. haematobium *negative 39 pg/ml (p = 0.0029) (Table 1, Figure 1). There was no difference in the median MIP-1α/CCL3 levels between HIV-1 positive and HIV-1 negative individuals (HIV negative 139 pg/ml, HIV positive 124 pg/ml, (p = 0.6312). In the 198 HIV-1 infected participants, MIP-1α/CCL3 concentration did not differ between participants with CD4 T-cell counts of 0.1 × 10^9^/L or more and those with counts below this value (Mann-Whitney test, p = 0.4993). There was no correlation between MIP-1α/CCL3 concentration and the CD4 T-cell counts (Spearman's r = 0.0039, p = 0.956).

**Figure 2:**
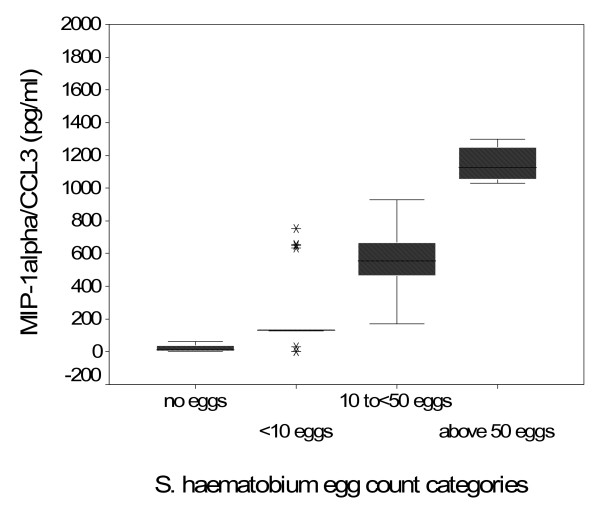
**Box-whisker plot of MIP-1α/CCL3 concentrations according to *S. haematobium *egg intensity, the classification is based on the WHO classification of; 0 = uninfected, < 10 eggs/10 ml of urine = light infection, 10 to <50 eggs/10 ml = moderate infection and more than 50 eggs/10 ml = high infection**. The egg infection intensity was determined by the urine filtration technique in 10 ml of urine collected over three consecutive days.

On accounting for the four schistosomiasis infection intensity groups, there was a statistically significant difference between the four infection groups, p = 0008, mainly due to the differences between individuals with *S. haematobium *infection and the uninfected. Also a statistically significant difference was observed in MIP-1α/CCL3 levels in an egg intensity-dependent manner, individuals with less than 10 eggs per 10 ml of urine having significantly lower MIP-1α/CCL3 than individuals with more than 10 eggs per 10 ml urine (Table 1, Figure 2). No difference in median MIP-1α/CCL3 concentration was observed between males and females, (males 127 pg/ml, females 144 pg/ml (p = 0.2133). Also no difference in median MIP-1α/CCL3 concentration was noted when the cohort was stratified into two age groups, below and above 25 years, p = 0.2570, and into five age groups, p = 0.3537 (Table 1).

**Figure 3:**
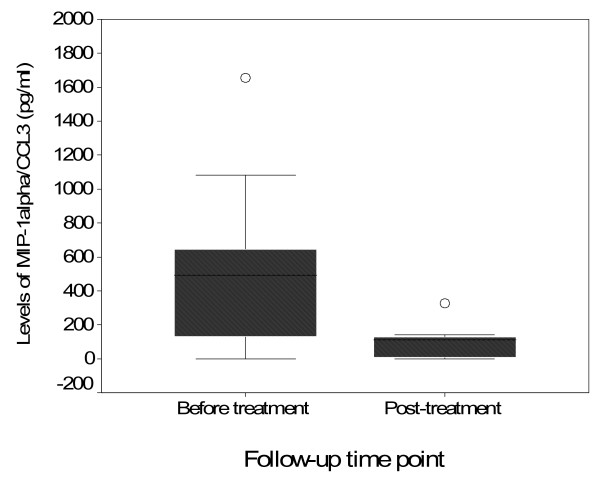
**Box-whisker plot of MIP-1α/CCL3 concentrations comparing baseline and three months post treatment with praziquantel**. The levels of MIP-1α/CCL3 were measured by ELISA using a standard curve within the detection range of 15 pg/ml - 2000 pg/ml.

Eighty-three (83) samples were analysed for MIP-1α/CCL3 at three months post treatment. Sixty one *S. haematobium *positive and 22 controls were selected for the post treatment analyses, only from individuals who received praziquantel treatment at baseline. Samples with high *S. haematobium *egg counts at baseline were prioritized for post treatment analysis, these were from individuals excreting more than 50 eggs/10 ml as heavy infection, within 10-50 eggs/10 ml as moderate infection intensity and from individuals excreting less than 10 eggs/10 ml as light infection. A comparison was done on the median MIP-1α/CCL3 concentration on samples from the same participants at baseline and at 3 months post treatment. The median MIP-1α/CCL3 concentration in plasma was significantly reduced at three months post treatment compared to baseline levels (Wilcoxon signed rank test, p = 0000, Figure 3).

## Discussion

Schistosomiasis and HIV-1 co-infection has been observed from a couple of studies in Zimbabwe and elsewhere to have far reaching consequences, especially in likelihood of immunopathology and AIDS progression [5-9]. However, no clear attributive aspects have been linked or are known to worsen the condition during co-infection. Generally, in addition to cytokines, chemokines also play an important role in the regulation of immune responses and in controlling infectious diseases including HIV infection and progression. Cytokine and chemokines are chemotactic for specific types of cells and are involved in immunoregulation of cell mediated immunity. MIP-1α/CCL3 play an important role in recruiting macrophages, dendritic cells and T cells to site of infection and lymphoid organs, a task perceived to likely contribute to the dangers of schistosomiasis and HIV-1 co-infection. In this study we found that schistosomiasis infection being depicted by high egg output significantly upregulated the plasma levels of MIP-1α/CCL3 irrespective of HIV-1 infection status. Some studies have identified MIP-1α/CCL3 as a marker of disease severity in *S. mansoni *infected individuals [11,25] and experimental studies in mice suggest that MIP-1α/CCL3 may be a causative factor in the development of severe schistosomiasis [24]. We investigated levels of MIP-1α/CCL3 in the plasma of HIV-1 and *S. haematobium *infected and uninfected individuals. These diseases are endemic in Zimbabwe where it is common to find individuals with HIV-1 and a parasitic co-infection.

From the observation on the co-infection data no differences in egg counts were found between HIV-1 positive participants and HIV-1 negative participants infected with *S. haematobium*. The findings were reported in detail by our group Kallestrup et al., (2005) [7]. However, briefly, the findings on the coinfection data on schistosomiasis egg counts reveal similarity between HIV-1 coinfected even after adjustments were made for differences in age and sex. These findings, led to speculation of an immunomodulatory inhibition of the human host's ability to excrete *Schistosoma *eggs when immunodeficient because of HIV-1 coinfection [35]. The findings indicated moderately to severely immunocompromised individuals whose interactions between HIV-1 and concurrent infections with *S. haematobium *indicated by higher egg counts cause inflammation during schistosomiasis. This finding is in agreement with other reports of an association between CD4 lymphocytopenia and helminthic and other infections [22,36-40]. These differences in CD4 cell counts apparently disappear in *S. mansoni *infected HIV-1 positive participants, indicating that any possible subtle effect of schistosomiasis on CD4 cell counts seems to be masked by the dramatic HIV-1 related decline in CD4 cell counts [35]. Despite the difference between the intensities of the *S. haematobium *infections in the population in the study and the *S. mansoni *infection in other studies, it appears that HIV-1 induced immunodeficiency does not impair the ability of participants with low and high intensity schistosomiasis to excrete *S. haematobium *eggs. Our results as first reported in Kallestrup et al., (2005) [7], further question the applicability of murine studies that show a dependency of adequate CD4 cell immunity for *S. mansoni *development and fecundity to human conditions. One may assume that, in our cohort, some antischistosome immune responses were already established when HIV-1 was encountered [7,22].

*Schistosoma haematobium *infected individuals had elevated levels of MIP-1α/CCL3 compared with egg negative individuals. This is in agreement with findings by other investigators on *S. mansoni *infection who demonstrated a positive correlation between elevated plasma concentrations of MIP-1α/CCL3, egg counts and presentation of severe schistosomiasis in humans [22-25]. Our results also showed that HIV-1 status had no influence on the levels of MIP-1α/CCL3 as individuals who were HIV-1 positive and *S. haematobium *positive had similar elevated levels as those who were HIV-1 negative and *S. haematobium *positive, suggesting that schistosomiasis infection is causing the elevation of MIP-1α/CCL3 levels. This is further confirmed by a comparison of MIP-1α/CCL3 levels in HIV-1 positive but *S. haematobium *negative compared to HIV-1 negative and *S. haematobium *negative, both groups showing low levels of MIP-1α/CCL3. Egg counts also had an influence on MIP-1α/CCL3 levels as shown by a comparison of the four groups which showed elevated levels in individuals with more than 10 eggs per 10 ml of urine compared to individuals with less than 10 eggs per 10 ml of urine. These findings indicated the existence of inflammatory environment during infection and egg laying by the schistosome worms. However, high egg output may be related to heavy worm burden subsequently related to increased inflammation by the high egg numbers released into the system.

We further determined the plasma concentration of MIP-1α/CCL3 at three months post treatment with the anti-schistosome drug, praziquantel. MIP-1α/CCL3 concentration in plasma was significantly reduced at three months post treatment, an indication that MIP-1α/CCL3 may be an inflammatory factor produced during *S. haematobium *infection and is downregulated when the infection is eliminated. Similar findings were reported in murine models [34]. It is assumed that eggs are the stimulant of this inflammatory response, and we may speculate that during inflammation, individuals co-infected with HIV-1 may be prone to immune activation and an increase in circulating viruses thereby increase chances of transmission. This calls for surveillance and control of schistosomiasis in population living in endemic areas, making praziquantel readily available for the control of schistosomiasis infection.

## Conclusion

The findings from this study have shown that the MIP-1α/CCL3 chemokine levels were associated with *S. haematobium *egg counts at baseline before treatment, but not with HIV-1 infection status. MIP-1α/CCL3 levels were significantly reduced at three months post treatment with praziquantel. We therefore conclude that MIP-1α/CCL3 chemokine is produced during inflammation, caused by eggs during *S haematobium *infection, in which infection is associated with increased MIP-1α/CCL3 levels in an egg intensity-dependent manner. Praziquantel treatment of *S. haematobium *is associated with a reduction in levels of MIP-1α/CCL3.

## Competing interests

The authors declare that they have no competing interests.

## Authors' contributions

RGZ, PK and EG conducted the field work. RGZ, EG, TM, SM and AEB designed and set the study objectives. RGZ and TM developed and conducted the immunoassays, analyzed the data and drafted the manuscript. RGZ, TM, EG, HU and EC designed the study field concept. All authors read the manuscript and approved the final version.

## Pre-publication history

The pre-publication history for this paper can be accessed here:

http://www.biomedcentral.com/1471-2334/9/174/prepub
